# Impact of preoperative diagnostic TURBT on progression-free survival in patients with pathological high-grade, stage T3/T4 bladder urothelial carcinoma

**DOI:** 10.18632/oncotarget.19159

**Published:** 2017-07-11

**Authors:** Haichao Huang, Zhenhua Liu, Xin Li, Wei Li, Jinchun Xing, Wei Yu, Jie Jin

**Affiliations:** ^1^ Department of Urology, The First Affiliated Hospital of Xiamen University, Siming District, Xiamen, Fujian 361003, China; ^2^ Department of Urology, Peking University First Hospital and Institute of Urology, Peking University, National Urological Cancer Center, Xicheng District, Beijing 100034, China

**Keywords:** diagnostic TURBT, cystoscopic biopsy, progression-free survival, urothelial carcinoma, bladder

## Abstract

Transurethral bladder tumor resection (TURBT) reportedly increases the circulating tumor cell count in patients with urothelial carcinoma of the bladder (UCB). To determine whether diagnostic TURBT leads to poorer progression-free survival (PFS) than diagnostic cystoscopic biopsy, we retrospectively reviewed the records of 96 consecutive primary pathological high-grade, stage T3/T4 UCB patients treated with radical cystectomy (RC) between January 2009 to December 2013. Clinicopathological features were collected from the medical records. PFS was determined from Kaplan-Meier curves, and potential independent prognostic factors for PFS were identified based on multivariable Cox analysis. During the follow-up period (median, 29 months), 43 patients experienced tumor progression (40 received diagnostic TURBT, 56 received cystoscopic biopsy). Patients who received cystoscopic biopsy had better PFS than those who received diagnostic TURBT (*p* = 0.008). Additionally, diagnostic TURBT was a significant risk factor for tumor metastasis in both univariable (HR: 2.219; 95% CI: 1.207–4.079; *p* = 0.010) and multivariable (HR: 2.455; 95% CI: 1.278–4.714; *p* = 0.007) Cox analyses. The present study provides the first evidence that diagnostic TURBT before RC negatively affects PFS in patients with pathological high-grade, stage T3/T4 UCB.

## INTRODUCTION

Bladder cancer is the most common malignancy of the urinary tract [1], and UCB is the most common pathological subtype. RC with pelvic lymph node dissection has been used as the standard therapy for muscle-invasive bladder cancer (MIBC), but the 5-year survival after RC for clinically localized MIBC is only approximately 50% [2]. The 5-year overall survival and tumor-specific survival for UCB patients with extravesical stage are only 30% and 37%, respectively [3–4]. The presence of circulating tumor cells (CTCs) has been confirmed to be associated with survival in several solid malignancies [5]. CTCs were detected in 17–50% of UCB patients and were significantly associated with tumor stage [6–8]. Diagnostic transurethral bladder tumor resection (TURBT) represents the standard initial, preoperative diagnostic procedure for MIBC. However, TURBT can damage the integrity of the bladder wall. Moreover, the pressure within the bladder during the process of TURBT would exceed the venous pressure, and it is impossible to completely resect the tumor for patients with extravesical stage (T3 and T4). Taken together, there is an increasing concern that TURBT may increase the risk of tumor metastasis. Recent studies revealed that TURBT may increase the count of CTCs for MIBC patients postoperatively [9–10]. Further studies are needed to determine whether TURBT increases the risk of metastatic diseases for patients with MIBC. Cystoscopic biopsy, another conventional initial diagnostic method for bladder cancer, seems to show less risk of metastatic events, as it causes relatively minor damage to the integrity of the bladder wall. In the present study, we evaluated whether preoperative diagnostic TURBT, compared with preoperative diagnostic cystoscopic biopsy, increased the risk of tumor progression for high-grade, stage T3/T4 UCB patients who underwent eventual RC.

## MATERIALS AND METHODS

### Patients and data collection

We retrospectively enrolled 96 patients with postoperative histological diagnosis as high-grade, stage T3/T4 UCB who underwent RC at two Chinese centers (Department of Urology, The First Affiliated Hospital of Xiamen University and Department of Urology, Peking University First Hospital) between January 2009 and December 2013. Patients with previous bladder tumors and other malignancies were excluded. Clinicopathological features, including age, gender, smoking history, method for obtaining preoperative histological evidence (diagnostic TURBT vs. cystoscopic biopsy), tumor size and number, tumor stage (T3 vs. T4), the presence of glandular differentiation, sarcomatoid differentiation, squamous differentiation, concomitant Carcinoma In Situ (CIS), lymphovascular invasion (LVI), prostatic urethra invasion (PUI), lymph node metastasis (LNM), post-operative adjuvant chemotherapy, and prognostic outcomes were collected. All patients received either diagnostic TURBT or diagnostic cystoscopic biopsy before the eventual RC.

### Treatment and follow-up

RC with pelvic lymph node dissection with or without postoperative adjuvant chemotherapy was applied as the main therapy for all patients within one month after the initial diagnostic TURBT or cystoscopic biopsy. The final pathological data analyzed in this study were based on post-RC standard pathological procedures. Tumor stage was assessed according to the Union for International Cancer Control (UICC) TNM classification of malignant tumors 2002. Tumor grade was assessed according to the WHO classification of 2004. The patients were seen every 6 months after RC. The follow-up visits consisted of a physical examination and ultrasonography. Other imaging analyses, including chest radiography and abdominal CT, were indicated if necessary. Local recurrences were defined as those occurring within the soft tissue field of exenteration, which is inside the bony pelvis. Distant recurrences were those occurring outside the pelvis [11–12]. Time to recurrence was defined as the period from the date of RC to the date when the first recurrence was diagnosed. Completed follow-up data were available for 86 patients.

### Statistical analysis

For statistical analysis, numerical variables with *t*-test and categorical variables with chi-square test were used for comparative analyses between diagnostic TURBT and cystoscopic biopsy groups. Survival curves were plotted using the Kaplan-Meier method, and significant differences were assessed using the Log-rank test. Subsequently, Cox regression analysis was applied to identify potential prognostic factors for survival. Characteristics that showed potential significance (*p* < 0.1) were further evaluated using multivariable Cox regression model. SPSS 22.0 was used in all statistical analyses. In all tests, *p* < 0.05 were considered to indicate significance. All experimental protocols were approved by Ethics Committee of Peking University First Hospital and Ethics Committee of The First Affiliated Hospital of Xiamen University. Informed consent was obtained from all subjects.

## RESULTS

### Baseline characteristics

A total of 96 high-grade, stage T3/T4 UCB patients were involved in our study; 27 patients were treated in the Department of Urology, The First Affiliated Hospital of Xiamen University, while the remaining 69 patients were treated in the Department of Urology, Peking University First Hospital. Based on the preoperative diagnostic methods, patients were grouped into the diagnostic cystoscopic biopsy group (*n* = 56) or the diagnostic TURBT group (*n* = 40). Analyses of these two cohorts demonstrated no statistically significant differences with regard to patient demographics, tumor number, tumor stage, the presence of glandular differentiation, sarcomatoid differentiation, squamous differentiation, concomitant CIS, LVI, PUI, LNM, or post-operative adjuvant chemotherapy status. However, there were significant differences with respect to tumor size between the two cohorts (89.3% with larger size for the biopsy group vs. 70.0% for the diagnostic TURBT group, *p* = 0.017) (Table 1). In addition, 37 patients received post-operative chemotherapy, among which 32 received gemcitabine/cisplatin (GC), 3 received gemcitabine/carboplatin, and 2 received gemcitabine/ paclitaxel regimen. The clinicopathological features of samples from the two Chinese centers are described in Table 2. Compared with patients selected from Peking University First Hospital, patients selected from The First Affiliated Hospital of Xiamen University had a larger proportion of patients aged 60 years or older (*p* = 0.011). No significant difference regarding the remaining clinicopathological features between samples from the two centers was observed.

**Table 1:** Descriptive clinicopathologic characteristics of patients treated with radical cystectomy and bilateral lymphadenectomy for pathological high-grade, stage T3/T4 urothelial carcinoma of the bladder

Characteristics	Cystoscopic biopsy, n (%) *N* = 56	Diagnostic TURBT, n (%) *N* = 40	Total	*P* value
Age (yr)				0.164
< 60	20 (35.7)	9 (22.5)	29	
≥ 60	36 (64.3)	31 (77.5)	67	
Gender				0.887
Male	47 (83.9)	34 (85.0)	81	
Female	9 (16.1)	6 (15.0)	15	
Smoking history				0.770
No	38 (67.9)	26 (65.0)	64	
Yes	18 (32.1)	14 (35.0)	32	
Tumor size (cm)				0.017
< 3	6 (10.7)	12 (30.0)	18	
≥ 3	50 (89.3)	28 (70.0)	78	
Tumor number (n)				0.558
< 3	36 (64.3)	28 (70.0)	64	
≥ 3	20 (35.7)	12 (30.0)	32	
pathologic stage				0.804
pT3	35 (62.5)	24 (60.0)	59	
pT4	21 (37.5)	16 (40.0)	37	
Concomitant CIS				0.426
Absent	44 (78.6)	34 (85.0)	78	
Present	12 (21.4)	6 (15.0)	18	
Squamous differentiation				0.744
Absent	36 (64.3)	27 (67.5)	63	
Present	20 (35.7)	13 (32.5)	33	
Sarcomatoid differentiation				1.000
Absent	49 (87.5)	35 (87.5)	84	
Present	7 (12.5)	5 (12.5)	12	
Glandular differentiation				0.844
Absent	41 (73.2)	30 (75.0)	71	
Present	15 (26.8)	10 (25.0)	25	
LVI				0.138
Absent	25 (44.6)	24 (60.0)	59	
Present	31 (55.4)	16 (40.0)	47	
PUI				0.102
Absent	33 (58.9)	30 (75.0)	63	
Present	23 (41.1)	10 (25.0)	33	
LN metastasis				0.129
Absent	43 (76.8)	25 (62.5)	68	
Present	13 (23.2)	15 (37.5)	28	
Postoperative adjuvant chemotherapy				0.127
No	38 (67.9)	21 (52.5)	59	
Yes	18 (32.1)	19 (47.5)	37	
Total	56 (58.3)	40 (41.7)	96	

CIS = Carcinoma In Situ; LVI = lymphovascular invasion; PUI = prostatic urethra invasion; LN = lymph node.

**Table 2:** Descriptive clinicopathologic characteristics of patients treated with radical cystectomy and bilateral lymphadenectomy for pathological high-grade, stage T3/T4 urothelial carcinoma of the bladder in two large tertiary referral centers of China

Characteristics	FAHXMU, *n* (%) *N* = 27	PKUFH, *n* (%) *N* = 69	Total	*P* value
Preoperative method				0.565
biopsy	17 (63.0)	39 (56.5)	56	
diagnostic TURBT	10 (37.0)	30 (43.5)	40	
Age (yr)				0.011
< 60	3 (11.1)	26 (22.5)	29	
≥ 60	24 (88.9)	43 (77.5)	67	
Gender				0.423
Male	21 (77.8)	60 (85.0)	81	
Female	6 (22.2)	9 (15.0)	15	
Smoking history				0.630
No	19 (70.4)	45 (65.2)	64	
Yes	8 (29.6)	24 (34.8)	32	
Tumor size (cm)				0.971
< 3	5 (18.5)	13 (18.8)	18	
≥ 3	22 (81.5)	56 (81.2)	78	
Tumor number (n)				0.630
< 3	19 (70.4)	45 (65.2)	64	
≥ 3	8 (29.6)	24 (34.8)	32	
pathologic stage				0.782
pT3	16 (59.3)	43 (62.3)	59	
pT4	11 (40.7)	26 (37.7)	37	
Concomitant CIS				0.260
Absent	20 (74.1)	58 (84.1)	78	
Present	7 (25.9)	11 (15.9)	18	
Squamous differentiation				0.411
Absent	16 (59.3)	47 (68.1)	63	
Present	11 (40.7)	22 (31.9)	33	
Sarcomatoid differentiation				0.198
Absent	26 (96.3)	58 (84.1)	84	
Present	1 (2.7)	11 (15.9)	12	
Glandular differentiation				0.616
Absent	19 (70.4)	52 (75.4)	71	
Present	8 (29.6)	17 (24.6)	25	
LVI				0.207
Absent	11 (40.8)	38 (55.1)	49	
Present	16 (59.2)	31 (44.9)	47	
PUI				0.893
Absent	18 (66.7)	45 (65.2)	63	
Present	9 (33.3)	24 (34.8)	33	
LN metastasis				0.151
Absent	22 (81.5)	46 (66.7)	68	
Present	5 (18.5)	23 (33.3)	28	
Postoperative adjuvant chemotherapy				0.112
No	20 (74.1)	39 (56.5)	59	
Yes	7 (25.9)	30 (43.5)	37	
Total	27 (28.1)	69 (71.9)	96	

FAHXMU = The First Affiliated Hospital of Xiamen University; PKUFH = Peking University First Hospital; CIS = Carcinoma In Situ; LVI = lymphovascular invasion; PUI = prostatic urethra invasion; LN = lymph node.

### Survival outcomes

**Figure 1:**
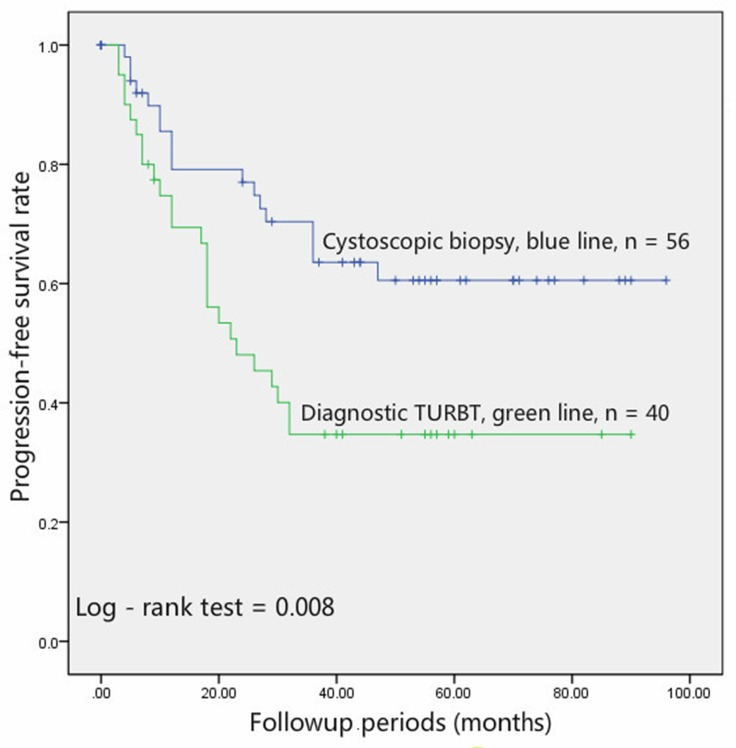
Patients undergoing diagnostic cystoscopic biopsy (blue line, *n* = 56) had a significantly (*p* = 0.008) better PFS than those undergoing diagnostic TURBT (green line, *n* = 40)

Completed follow-up data were available for 86 patients, with a median follow-up period of 29 months. During the follow-up period, 43 patients experienced disease progression, 18 from the preoperative diagnostic cystoscopic biopsy group and 25 from the preoperative diagnostic TURBT group. Survival data displayed in Kaplan-Meier curves demonstrated significant differences with respect to PFS between diagnostic TURBT and cystoscopic biopsy groups (*p* = 0.008, Figure 1). The 3-year PFS rates for patients in the diagnostic biopsy and TURBT groups were 63.6% and 48.1%, respectively.

In the univariable Cox analysis, preoperative diagnostic TURBT, smoking history, the presence of sarcomatoid differentiation, LNM, and higher pathological stage (pT4) were significantly associated with an unfavorable PFS. However, only preoperative diagnostic TURBT, smoking history, the presence of sarcomatoid differentiation and higher pathological stage (pT4) retained the significant association with survival following the multivariable analysis (Table 3).

**Table 3:** Univariate and multivariate Cox regression analyses for prediction of progression-free survival in 96 patients treated with radical cystectomy and bilateral lymphadenectomy for high-grade, stage T3/T4 urothelial carcinoma of the bladder

Univariate analyses	HR	95% CI	*P*
TURBT before RC	2.219	1.207–4.079	0.010
Postoperative adjuvant chemotherapy (yes)	0.792	0.427–1.471	0.461
Smoking history (+)	1.670	0.912–3.055	0.096
Tumor size (≥ 3 cm)	0.876	0.406–1.890	0.736
Tumor number (≥ 3 cm)	1.160	0.625–2.154	0.638
Male gender	1.987	0.710–5.563	0.191
Age (≥ 60 yr)	1.416	0.712–2.817	0.322
Sarcomatoid differentiation (present)	1.915	0.887–4.137	0.098
Squamous differentiation (present)	0.973	0.507–1.866	0.934
Glandular differentiation (present)	0.784	0.395–1.556	0.486
PUI (+)	1.096	0.590–2.036	0.771
LVI (+)	0.872	0.478–1.593	0.656
Concomitant CIS (present)	0.686	0.289–1.626	0.392
LN metastasis (+)	1.997	1.074–3.712	0.029
Pathologic tumor stage (T4)	1.830	1.006–3.331	0.048

Multivariate analyses	HR	95% CI	*P*
TURBT before RC	2.455	1.278–4.714	0.007
Smoking history (+)	1.958	1.040–3.686	0.037
Sarcomatoid differentiation (present)	2.965	1.285–6.843	0.011
LN metastasis (+)	1.389	0.715–2.698	0.332
Pathologic tumor stage (T4)	2.004	1.085–3.703	0.026

CIS = Carcinoma In Situ; LVI = lymphovascular invasion; PUI = prostatic urethra invasion; LN = lymph node.

## DISCUSSION

Bladder cancer is the most common malignancy of the urinary tract [1]. On average, 70% of bladder tumors present as non-muscle-invasive bladder cancer (NMIBC), the remaining 30% as MIBC. For the MIBC group, LNM occurs in approximately 25% patients [6]. Moreover, although RC represents the current gold standard therapy for MIBC, distant metastasis occurs in 50% of MIBC patients within 2 years [13]. CTCs are cancer cells which originate from the primary tumor site and invade the lymphatics and systemic circulation during the initial sequences of metastatic progression [14–15]. While the majority of CTCs die in the bloodstream, a few cells eventually form new colonies at distant sites. Thus, the presence of CTCs may indicate micrometastasis, which represents an early stage of the metastatic process. In addition, CTCs, which are associated with tumor recurrence and metastasis in other solid malignancies (including, breast, rectum, and prostate cancer), have emerged as a potential candidate to explain the high progression rate of MIBC [5].

Recent studies showed that TURBT increases the count of CTCs for UCB patients, especially for the MIBC population. Engilbertsson and colleagues [9] evaluated 16 UCB patients (10 MIBC, 6 NMIBC) who underwent TURBT, from which 6 (5 MIBC, 1 NMIBC) positive inferior vena cava samples were found to have an increased number of CTCs intraoperatively. In addition, Blaschke and colleagues reported a similar result recently, where the CTC count increased postoperatively in all of the T2 high-grade UCB patients [10]. However, Antoniewicz and colleagues [16] evaluated CTC count of 51 UCB patients. In their study, no significant evidence for increased level of circulating urothelial cells in the peripheral blood after TURBT was found. Interestingly, half of their included patients had NMIBC. Taken together, MIBC patients, compared with NMIBC patients, are more likely to be affected by the TURBT. Moreover, in the systematic review and meta-analysis of Msaouel and Koutsilieris [7], CTC-negative patients compared favorably to CTC-positive patients with respect to the proportion of advanced (stage III-IV) disease. Thus, further studies are clearly required to ascertain whether diagnostic TURBT increases the risk of metastatic diseases for UCB patients with extravesical pathological stage (T3 and T4).

Both TURBT and cystoscopic biopsy are performed to obtain histologic evidence for patients with suspicion of UCB in daily clinical work. Diagnostic TURBT provides more pathological information than cystoscopic biopsy, which is of great help in pathological staging diagnosis and therapeutic decision-making. However, compared with cystoscopic biopsy, TURBT makes larger wounds in the bladder wall, induces higher level of pressure within the bladder, and causes some complications especially for T3–4 patients, such as bladder perforation. Moreover, diagnostic TURBT costs more in medical fees than diagnostic cystoscopic biopsy. Thus, patients who received cystoscopic biopsy as the diagnostic surgical approach before RC were identified as the control group, comparing the oncologic outcomes with those received diagnostic TURBT before RC and verifying the impact of diagnostic TURBT in tumor metastasis for high-grade, stage T3/T4 UCB. In addition, although CT and MRI have limited capability to detect microscopic invasion of the perivesical fat, it may be used to find T3b disease or higher with greater diagnostic accuracy [17]. Taken together, some surgeons of our centers were inclined to choose diagnostic cystoscopic biopsy for patients with clinical T3 and T4 stage confirmed by CT scans before eventual RC. Considering the retrospective feature of our study, whether a patient received diagnostic TURBT or cystoscopic biopsy was based on surgeon–patient decision rather than other standards. In addition, we found that there were more patients with larger tumor size in the biopsy group than in the TURBT group in our cohort (*p* = 0.017), suggesting that tumor size was an important factor that was considered in making the surgeon–patient decision. In other words, when a larger tumor was detected, the biopsy, rather than the diagnostic TURBT, was more likely to be performed.

In the survival analysis, patients who received cystoscopic biopsy showed a longer PFS than those underwent diagnostic TURBT (*p* = 0.008, Figure 1). In addition, the univariable and multivariable Cox analysis showed that diagnostic TURBT increased the risk the tumor metastasis (*p* = 0.010 and 0.007, respectively). Our study suggests that diagnostic TURBT may increase the risk of tumor metastasis and compromise the PFS for high-grade, stage T3/T4 UCB patients. The potential mechanisms by which diagnostic TURBT compromises the PFS of high-grade, stage T3/T4 UCB may be attributed to the fact that the process of TURBT increases the level of CTCs and induces micrometastasis. To the best of our knowledge, we are the first to provide the evidence that diagnostic TURBT, using patients underwent cystoscopic biopsy before RC as the control group, may induce a poor PFS of high-grade, stage T3/T4 UCB patients.

The current study was limited by the inherent retrospective feature, which makes it impossible to evaluate the differential impact between TURBT and biopsy in increasing the degree of CTCs count. Another limitation lies in the fact that none of the patients enrolled in the current study received neoadjuvant chemotherapy (NAC). Indeed, NAC could improve the survival of patients with MIBC. However, no molecular profiling of the tumor is now available to identify responders, which will lead to overtreatment of nonresponders. In addition, delayed cystectomy in nonresponders may compromise final outcome. Thus, even in the majority of American and European clinical practice, the utilization rate of NAC was low [18–22]. In our centers, though NAC was recommended to patients, no patient decided to undergo NAC before the RC, which can be attributed to the concerns mentioned above. Furthermore, as we only included tumors with extravesical stage (pathological stage T3 and T4), whether diagnostic TURBT remains as a risk factor for tumor metastasis for tumors with organ-confined stage (stage T1–2) is unknown. Other Limitations of our investigation include the relatively small sample size and short follow-up period. However, for tumors with this pathological stage, the majority of progression events would occur within 2 years after the RC.

## CONCLUSIONS

In conclusion, our results provide the first evidence that diagnostic TURBT before RC may induce an unfavourable PFS for patients with pathological high-grade, stage T3/T4 UCB. However, further prospective, randomized studies with larger sample size and longer follow-up period are needed.

## Abbreviations

TURBT: transurethral bladder tumor resection
UCB: urothelial carcinoma of bladder
PFS: progression-free survival
RC: radical cystectomy
MIBC: muscle invasive bladder cancer
CTCs: circulating tumor cells
CIS: concomitant Carcinoma *In Situ*
PUI: prostatic urethra invasion
LVI: lymphovascular invasion
LNM: lymph node metastasis
NAC: neoadjuvant chemotherapy
NMIBC: non-muscle invasive bladder cancer

## References

[1] Siegel R, Ma J, Zou Z, Jemal A (2014). Global cancer statistics, 2014. CA Cancer J Clin.

[2] Stein JP, Skinner DG (2006). Radical cystectomy for invasive bladder cancer: long-term results of a standard procedure. World J Urol.

[3] Gschwend JE, Dahm P, Fair WR (2002). Disease specific survival as endpoint of outcome for bladder cancer patients following radical cystectomy. Eur Urol.

[4] Stein JP, Lieskovsky G, Cote R, Groshen S, Feng AC, Boyd S, Skinner E, Bochner B, Thangathurai D, Mikhail M, Raghavan D, Skinner DG (2001). Radical cystectomy in the treatment of invasive bladder cancer: long-term results in 1054 patients. J Clin Oncol.

[5] Maheswaran S, Haber DA (2010). Circulating tumor cells: a window into cancer biology and metastasis. Curr Opin Genet Dev.

[6] Pashos CL, Botteman MF, Laskin BL, Redaelli A (2002). Bladder cancer: epidemiology, diagnosis, and management. Cancer pract.

[7] Msaouel P, Koutsilieris M (2011). Diagnostic value of circulating tumor cell detection in bladder and urothelial cancer: systematic review and meta-analysis. BMC cancer.

[8] Flaig TW, Wilson S, van Bokhoven A, Varella-Garcia M, Wolfe P, Maroni P, Genova EE, Morales D, Lucia MS (2011). Detection of circulating tumor cells in metastatic and clinically localized urothelial carcinoma. Urology.

[9] Engilbertsson H, Aaltonen KE, Björnsson S, Kristmundsson T, Patschan O, Rydén L, Gudjonsson S (2015). Transurethral bladder tumor resection can cause seeding of cancer cells into the bloodstream. J Urol.

[10] Blaschke S, Koenig F, Schostak M (2016). Hematogenous tumor cell spread following standard transurethral resection of bladder carcinoma. Eur Urol.

[11] Stein JP, Lieskovsky G, Cote R, Groshen S, Feng AC, Boyd S, Skinner E, Bochner B, Thangathurai D, Mikhail M, Raghavan D, Skinner DG (2001). Radical cystectomy in the treatment of invasive bladder cancer: long-term results in 1,054 patients. J Clin Oncol.

[12] Mitra AP, Quinn DI, Dorff TB, Skinner EC, Schuckman AK, Miranda G, Gill IS, Daneshmand S (2010). Factors influencing post-recurrence survival in bladder cancer following radical cystectomy. BJU Int.

[13] Karl A, Carroll PR, Gschwend JE, Knüchel R, Montorsi F, Stief CG, Studer UE (2009). The impact of lymphadenectomy and lymph node metastasis on the outcomes of radical cystectomy for bladder cancer. Eur Urol.

[14] Mocellin S, Keilholz U, Rossi CR, Nitti D (2006). Circulating tumor cells: the ‘leukemic phase’ of solid cancers. Trends Mol Med.

[15] Msaouel P, Pissimissis N, Halapas A, Koutsilieris M (2008). Mechanisms of bone metastasis in prostate cancer: clinical implications. Best Pract Res Clin Endocrinol Metab.

[16] Antoniewicz AA, Paziewska A, Mikula M, Goryca K, Dabrowska M, Poletajew S, Borowka A, Ostrowski J (2012). Lack of evidence for increased level of circulating urothelial cells in the peripheral blood after transurethral resection of bladder tumors. Int Urol Nephrol.

[17] Rajesh A, Sokhi HK, Fung R, Mulcahy KA, Bankart MJ (2011). Bladder cancer: evaluation of staging accuracy using dynamic MRI. Clin Radiol.

[18] Porter MP, Kerrigan MC, Donato BM, Ramsey SD (2011). Patterns of use of systemic chemotherapy for Medicare beneficiaries with urothelial bladder cancer. Urol Oncol.

[19] Zaid HB, Patel SG, Stimson CJ, Resnick MJ, Cookson MS, Barocas DA, Chang SS (2014). Trends in the utilization of neoadjuvant chemotherapy in muscle-invasive bladder cancer: results from the National Cancer Database. Urology.

[20] Sfakianos JP, Galsky MD (2015). Neoadjuvant chemotherapy in the management of muscle- invasive bladder cancer bridging the gap between evidence and practice. Urol Clin N Am.

[21] Schiffmann J, Sun M, Gandaglia G, Tian Z, Popa I, Larcher A, Meskawi M, Briganti A, McCormack M, Shariat SF, Montorsi F, Graefen M, Saad F, Karakiewicz PI (2016). Suboptimal use of neoadjuvant chemotherapy in radical cystectomy patients: A population-based study. Can Urol Assoc J.

[22] Reardon ZD, Patel SG, Zaid HB, Stimson CJ, Resnick MJ, Keegan KA, Barocas DA, Chang SS, Cookson MS (2015). Trends in the Use of Perioperative Chemotherapy for Localized and Locally Advanced Muscle-invasive Bladder Cancer: A Sign of Changing Tides. Euro Urol.

